# B Cells Control Mucosal-Associated Invariant T Cell Responses to *Salmonella enterica* Serovar Typhi Infection Through the CD85j HLA-G Receptor

**DOI:** 10.3389/fimmu.2021.728685

**Published:** 2021-10-01

**Authors:** Rosângela Salerno-Gonçalves, Tasmia Rezwan, David Luo, Hervé Tettelin, Marcelo B. Sztein

**Affiliations:** ^1^ Center for Vaccine Development and Global Health (CVD), Department of Pediatrics, University of Maryland School of Medicine, Baltimore, MD, United States; ^2^ Department of Microbiology and Immunology and Institute for Genome Sciences (IGS), University of Maryland School of Medicine, Baltimore, MD, United States; ^3^ Program in Oncology, University of Maryland Marlene and Stewart Greenebaum Comprehensive Cancer Center, Baltimore, MD, United States

**Keywords:** *S*. Typhi, MAIT cells, HLA-G, intestine, human

## Abstract

Mucosal-associated invariant T (MAIT) cells are an innate-like population of T cells that display a TCR Vα7.2+ CD161+ phenotype and are restricted by the nonclassical MHC-related molecule 1 (MR1). Although B cells control MAIT cell development and function, little is known about the mechanisms underlying their interaction(s). Here, we report, for the first time, that during *Salmonella enterica* serovar Typhi (*S*. Typhi) infection, HLA-G expression on B cells downregulates IFN-γ production by MAIT cells. In contrast, blocking HLA-G expression on *S*. Typhi-infected B cells increases IFN-γ production by MAIT cells. After interacting with MAIT cells, kinetic studies show that B cells upregulate HLA-G expression and downregulate the inhibitory HLA-G receptor CD85j on MAIT cells resulting in their loss. These results provide a new role for HLA-G as a negative feedback loop by which B cells control MAIT cell responses to antigens.

## Introduction

MAIT cells are innate-like T-cells restricted by the nonclassical MHC-related molecule 1 (MR1) that exhibit a TCR Vα7.2+ CD161+ phenotype (1–4). MAIT cells have broad anti-microbial reactivity and can mediate strong immune responses to bacteria such as *Mycobacterium tuberculosis* (Mtb), *Vibrio cholera*, *Escherichia coli* (*E. coli*), *Shigella, Helicobacter pylori*, *S.* Typhimurium, *S*. Paratyphi A, and *S*. Typhi (1, 5–11).

B cells are major antigen-presenting cells during the initial (12) and re-stimulation phases (13) of host immune responses. *Salmonella*-infected B cells can effectively re-stimulate CD4+ cells and classical and nonclassical CD8+ T-cells (9, 10, 13–23). Activated B cells express the Lectin-like transcript-1 (LLT1) molecule, the CD161 ligand on the MAIT cell membrane surface (24–27). CD161 is expressed in all human T cells capable of producing IFN-γ and IL-17A, including MAIT, CD4+, and CD8+, whether the express T cell receptor (TCR)αβ or TCRγδ (7, 27–31). Moreover, depletion of B cells with anti-CD20 antibodies showed that B cells regulate CD8+ T cell differentiation (32). Indeed, a seminal study from Kaufmann’s research team showed the involvement of B cell responses in the control of *Salmonella* typhimurium infection in mice that completely lack antibodies (33). For instance, B cell-intrinsic MyD88 signaling, and B cell receptor-mediated antigen presentation are necessary for developing Th1 responses to *Salmonella* (34, 35). Moreover, previous observations from McSorley’s group showed in a mouse model that deficiency of B cell-dependent antigen presentation, but not their antibodies, had a major role in the reductions of CD4 T cell IFN-γ production and susceptibility to *Salmonella* infection (36). Mastroeni and colleagues also found that transfer of immune serum did not restore impaired Th1 T-cell responses to *Salmonella* antigens in B cell-deficient mice (37). Despite these observations, little is known regarding the mechanisms by which B cells stimulate or inhibit MAIT cell function during *S*. Typhi infection.

Our group recently reported the first direct evidence that CD8+ MAIT cells are activated, secrete cytokines, and kill B cells exposed to gut commensals and pathogens, such as *S*. Typhi (9). The cytokine profile of these responses was dependent on bacterial load but independent of the expression level of MR1 or bacterial antigen on B cell surfaces (9). Our group also demonstrated that bacterial load played an essential role in activating and homing characteristics of CD8+ MAIT cells after a human experimental challenge with wild-type *S*. Typhi (10). MAIT cells from volunteers susceptible to typhoid disease after receiving a low-dose inoculum of *S*. Typhi had higher levels of CD38 co-expressing CCR9, CCR6, and Ki67 during the development of typhoid fever than individuals receiving a high-dose inoculum (10). MAIT cell development is a multi-step process involving an intra-thymic selection process dependent on MR1 but independent of B cells or commensal flora (38, 39). This step is followed by a peripheral maturation and expansion that requires both B cells and commensal flora (3, 38). Human B cells can also control expression of Th1 and T follicular helper signatures, which support cellular and humoral immunity (40). On the other hand, MAIT cells have the capacity for helping B cells by increasing the secretion of cytokines/chemokines associated with B cell activation, migration, and regulation of antibody production (41–45). Thus, we hypothesize that in the periphery, human B cells, through a feedback mechanism, can modify MAIT cell responses to *S*. Typhi by modulating the expression of defined molecules.

Here, we showed for the first time that by a feedback loop involving the CD85j HLA-G receptor, B cells modulate IFN-γ production by MAIT cells. These results will guide future investigations in the study of a new phenomenon, i.e., the study of regulatory mechanisms involving HLA-G and CD85j in modulating MAIT cell responses to infections/pathogens.

## Materials and Methods

### Ethics Statement

The human experimentation guidelines of the US Department of Health and Human Services and those of the University of Maryland, Baltimore, were followed in the present clinical research. Volunteers enrolled in the University of Maryland Institutional Review Board protocol number HP-00040025 gave signed consent authorizing the collection of blood specimens for the studies included in this manuscript.

### Subjects

Nine healthy volunteers, 20-59 years old, recruited from the Baltimore-Washington area and the University of Maryland at Baltimore campus, participated in this study. Volunteers were screened for good health by medical history, physical examination, and routine laboratory tests, including blood counts and the absence of antibiotic treatment. Peripheral blood mononuclear cells (PBMC) were isolated by density gradient centrifugation and cryopreserved in liquid N_2_.

### B-LCLs, *S*. Typhi Infection, and Co-Culture

B-LCLs were incubated in RPMI in a 37°C 5% CO_2_ incubator with wild-type *S*. Typhi strain Ty2 at different cell:bacteria multiplicity of infection (MOI) (18) for 3 hours. Cells left uninfected were used as controls. *S*. Typhi strain Ty2 was obtained through the cryobank at the Center Vaccine Development and Global Health (CVD). After 16-18 hours of gentamicin treatment, B-LCLs were irradiated (6,000 rads), and surface stained with a monoclonal antibody (mAb) to CD45, a marker abundantly expressed on the surface of hematopoietic cells (46). After staining, autologous B-LCL were co-cultured with overnight-rested PBMC at a B-LCL : PBMC cell ratio of 1:5 in R10 media (i.e., RPMI 1640 (Gibco, Grand Island, New York) media supplemented with 100 U/mL penicillin, 100 µg/mL streptomycin, 50 µg/mL gentamicin, 2 mM L-glutamine, 1 mM sodium pyruvate, 10 mM HEPES (Gibco) buffer and 10% heat-inactivated fetal bovine serum). When noted, human recombinant IFN-γ (Sigma St. Louis, MO, USA) was added to the co-culture at 0.5, 5, or 25 ng/ml, Time-course experiments were used to track phenotypic changes (3, 6, and 16 hours). Subsequently, cells were labeled and evaluated by conventional flow cytometry or mass cytometry as described below.

### Primary B Cell Isolation

Primary B cells were isolated as previously described (9). Briefly, B cells were isolated from PBMC by negative selection using an immunomagnetic bead kit (Invitrogen) resulting in untouched (i.e., non-activated) human B cells. Assay was performed as per manufacturer’s instructions. B cell populations were ~90% pure as determined by flow cytometric analysis.

### Sorted MAIT Cells and Co-Culture Experiment

MAIT cells (CD3+CD4-CD14-CD19-IL-18R+ TCR Va7.2+) were sorted from PBMC by using a MoFlo Astrios EQ (Beckman Coulter, Miami, FL, USA) cell sorter system. Sorted MAITs were >95% pure as determined by flow cytometric analysis. Approximately 100,000 of sorted MAIT cells were co-cultured for 16-20 hours with 40,000 of B-LCL at a B-LCL : PBMC cell ratio of 1:2.5 in R10 media. B-LCL were prepared as described above (“B-LCLs, *S.* Typhi infection, and co-culture” section).

### Blocking Experiments

Blocking experiments were performed using purified anti-human mAbs to CD161 (clone DX12, BD Pharmingen, San Diego, CA), and HLA-G (clone 87G, Biolegend, San Diego, CA), and polyclonal goat antibodies to Lectin-Like Transcript-1 (LLT-1) (R&D Systems, Minneapolis, MN). To block CD161 expressed by MAIT cells, PBMC were preincubated for 1 hour with anti-CD161 or purified mouse IgG1, κ isotype control mAbs (20 μg/ml) before being co-cultured with CD45 labeled B-LCL. To block LLT1, B-LCLs were preincubated for 1 hour with anti-LLT1 or purified normal goat IgG control (5 and 20 μg/ml) before co-culture with MAIT cells. To block HLA-G, B-LCLs were preincubated for 1 hour with purified anti-human HLA-G, or purified mouse IgG2a, κ Isotype control before co-culture with MAIT cells. After blocking and without washing, B-LCLs and MAIT cells were co-cultured as described below in the “flow and mass cytometry” sections. Experiments were performed to determine the optimal timing and dilution of the HLA-G blocking antibodies. HLA-G mAbs were tested at 1, 2.5, 5, and 25 μg/ml, and the optimal concentration was identified as 2.5 μg/ml for HLA-G. Effect of HLA-G blocking was accessed after 3 and 16 hours of coculture, and optimal timing determined to be after 16 hours.

### Fluorochrome-Labeled Antibodies

Cells were stained with mAbs to CD3 (clone UCHT1), CD69 (clone TPI-55-3) (Beckman-Coulter, Miami, FL), CD8 (clone HIT8a), CD161 (clone DX12), IL-10 (clone JES3-9D7), interferon (IFN)-γ (clone B27), tumor necrosis factor (TNF)-α (clone MAb11) (BD Pharmingen, San Diego, CA, USA), CD14 (clone TuK4), CD45 (clone H130) (Invitrogen, Carlsbad, CA), and HLA-G (clone 87G), CD85j (ILT2) (clone GHI/75), and TCRα7.2 (clone 3C10)(Biolegend, San Diego, CA). Polyclonal antibody to the *Salmonella* Common Structural Antigens (CSA) (KPL, Gaithersburg, MD, USA) was used to measure *S.* Typhi infection. Antibodies conjugated to the following fluorochromes were used in these studies: Fluorescein isothiocyanate (FITC), Phycoerythrin (PE), PE-Cy5, Peridinin chlorophyll protein (PerCP)-Cy5.5, PE-Cy7, Pacific Blue, Brilliant Violet (BV) 570, BV605, BV650, Quantum dot (QD) 800, Alexa 647, allophycocyanin (APC)-Alexa 700, or APC-H7.

### Flow Cytometry

Flow cytometry assays were performed as previously described with small modifications (9, 10). Briefly, CD45-stained-B-LCL cells were co-cultured with PBMC containing MAIT cells, and stained with a dead-cell discriminator, violet or yellow fluorescent viability dye (ViViD or YeViD Invitrogen), followed by two successive 30 minute-incubations with human IgG to block Fc receptors, and a fluorochrome-labeled antibody cocktail for surface markers (CD3, CD8, CD14, TCRα 7.2, CD161, HLA-G, CD85j, and CSA). Cells were then fixed, permeabilized, and intracellularly stained with mAbs specific to IL-10, IFN-γ, or TNF-α. Cells were analyzed by flow cytometry on an LSR-II instrument (BD Biosciences). Data were evaluated with WinList v7.0 (Verity Software House, Topsham, ME). Uninfected and unstimulated cells were used to assess nonspecific binding to B-LCL and MAIT cells, respectively. Lymphocytes were gated based on their light scatter characteristics. Single lymphocytes were gated based on forward scatter height *vs.* forward scatter area. To gate MAIT cells, a “dump” channel was used to eliminate dead cells (ViViD^+^/YeViD+) as well as macrophages/monocytes (CD14^+^) and B-LCLs (CD45^+^) from the analysis. This was followed by additional gating on CD3, CD8, TCR Vα7.2, CD161 to identify cytokine-producing (IFN-γ) MAIT cells. To gate B-LCLs, after elimination of dead cells (ViViD^+^/YeViD+), cells were gated on CD3 negative population followed by CD45 to identify B-LCLs.

### Metal-Labeled Antibodies

Cells were stained with anti-human mAbs to CD3 (clone UCHT1), CD4 (clone SK3), CD8 (clone RPA-T8), CD16 (clone 3G8), CD19 (clone HIB19), CD45 (clone HI30), CD69 (clone FN50), CD161 (clone HP-3G10), IFN-γ (clone B27) (Fluidigm, Sunnyvale, CA), CD14 (clone 3C10) (Invitrogen, Carlsbad, CA), CD107a (clone H4A3), CD107b (clone H4B4), HLA-G (clone 87G), IL-10 (clone JES3-9D7), TNF-α (clone MAb11) and TCR Vα7.2 (clone 3C10) (Biolegend, San Diego, CA), and polyclonal CSA (KPL). All antibodies, other than the ones purchased from Fluidigm, were metal labeled with MaxPar X8 labeling kits (Fluidigm) according to the manufacturer’s instructions.

### Mass Cytometry

Mass cytometry assays were performed as previously described with small modifications (47). Briefly, CD45-stained-B-LCL cells were co-cultured with PBMC containing MAIT cells in the presence of mAbs to CD107a and CD107b (15 μl of each/1 x 10^6^ cells in 500 μl of R10 medium). After 3 or 16 hours, cells were harvested and stained for live/dead cell with Cisplatin (198 Pt), followed by two successive 30 minute-incubations with human IgG to block Fc receptors, and a metal-labeled antibody cocktail for surface markers to identify cell lineages (*e.g*., B and MAIT cells, as described by flow cytometry), HLA-G expression and infection (CSA). Cells were then fixed, permeabilized, and intracellularly stained with mAbs specific to cytokines (*e.g*., IFN-γ; TNF-α, and IL-10). Finally, samples were stained with 103Rh for cell DNA detection by mass cytometry. Cells were resuspended in EQ4 normalization beads and acquired within 48 hours using a mass cytometer. Single cells were gated for Gaussian parameters (Event length, Centre, Residual, and Width values), DNA high (103Rh), and live (Cisplatin low; 198Pt) parameters. After dead/live discrimination, the remaining gating strategies were similar to those applied for flow cytometry. The acquisition was performed using a Helios mass cytometer (Fluidigm), and data evaluated *via* Cytobank (Santa Clara, CA), a cloud-based platform. Uninfected and unstimulated cells were used to assess nonspecific binding to B-LCL and MAIT cells, respectively.

### IFN-γ Production

IFN-γ production was measured using the Meso Scale Discovery platform (MSD, Gaithersburg, MD)-assay and carried out following the manufacturer’s instructions. After 16-18 hours of B-LCL and MAIT cell co-culture, supernatants were harvested and kept at -20°C until assayed.

### Quantification of HLA-G and LLT-1 Transcripts by Real-Time Quantitative PCR (qRT-PCR)

Total RNA isolation and qRT-PCR were performed as previously described with a few modifications (48, 49). Briefly, total cellular RNA was isolated from 3 x 10^6^ cells using the RNeasy Mini Isolation Kit (Qiagen, Valencia, CA), and RNA concentrations measured on a NanoDrop 1000 spectrophotometer. Subsequently, 1.0 μg of RNA was treated with DNase I (Qiagen) and used for cDNA synthesis with RT2 first strand Reverse Transcription Kit (Qiagen). A total of 20-50 ng of cDNA was used per qRT-PCR, and amplified material was detected using RT² SYBR^®^ Green qPCR Mastermix (Qiagen). qRT-PCR was performed on ABI 7900HT thermocycler (Applied Biosystems), with cycling conditions of 95°C for 10 min, 40 cycles at 95°C for 15 sec and 60°C for 1 min. After amplification, PCR product sizes were inspected on a 1.0% agarose gel, and clear bands with the estimated amplicon sizes were seen. The relative levels of the HLA-G (50) (forward primer: 5’-GCTGCCCTGTGTGGGACTGAGTG; reverse primer: 5’- GACGGAGACATCCCAGCCCCTTT), and LLT1 (51) (forward primer: 5’-TTCCTATCCTGGGAGCAGGA; reverse primer: 5’- GACATGTATATCTGATTTGGAACAA) gene were normalized to the GAPDH housekeeping gene (forward primer: 5’-GAAGGTGAAGGTCGGAGT; reverse primer: 5’-GAAGATGGTGATGGGATTTC).

### Statistical Analysis

All statistical tests were performed using Prism software (version 7, GraphPad Software, La Jolla, CA). Comparison between two groups were performed by two-sided paired Student’s t-test. Comparison between multiple groups applied one-way ANOVA were performed with Tukey’s *posthoc* analysis. Correlations utilized the Pearson Product Moment tests. *P* values <0.05 were considered significant. Principal Component Analysis (PCA) was performed as previously described (47, 52) using the cloud-based platform ClustVis web tools (53). Briefly, unit variance scaling was applied to rows, and singular value decomposition (SVD) with imputation used to calculate principal components.

## Results

### Decrease in the Percentage of MAIT Cells Expressing CD161 Molecules After Exposure to Bacteria Infected B Cells

Our group previously investigated the role of cell-to-cell contact in MAIT cell activation by performing experiments using transwell filters (9). In these experiments, we used B-lymphoblastoid cell lines (B-LCLs) as target cells. B-LCLs were generated by infecting PBMC with Epstein Barr Virus (EBV), which has been demonstrated to immortalize human resting B cells *in vitro*, giving rise to an actively proliferating B cell population (54). B-LCLs bear negligible genetic and phenotypic alterations from their counterpart B cells (55–58). We found that MAIT cells in contact with autologous *S*. Typhi-infected B-LCL increased both the percentage of the early activation marker CD69 and the production of IFN-γ (9). In contrast, marginal or no increases were observed when MAIT cells were alone, in contact with uninfected B-LCLs, or separated from infected B-LCLs by transwell filters (9). Thus, B cell-MAIT cell interactions are required to shape MAIT cell functions. Besides this information, little is known regarding how B cells regulate or promote these MAIT cell functions. To further understand B cell-MAIT cell interactions, PBMC were cultured alone (none) or co-cultured with autologous B-LCLs that were left uninfected or infected with *S*. Typhi at 1:10 multiplicity of infection (MOI). After 16-18 hours of incubation, cells were collected and labeled using an antibody panel, including CD3, CD8, IL-18R, TCR Vα7.2, and CD161. In agreement with previous studies (59), we observed significant decreases in the percentage of MAIT cells when co-cultured with *S*. Typhi-infected (INF) B-LCLs, as compared to those cultured alone (none) or co-cultured with uninfected (UN) B-LCLs (**Figures S1A, B**). To determine whether these decreases were, at least in part, due to a reduction of CD161 expressing MAIT cells, we compared CD161 expression in IL-18R+ TCR Vα7.2+ MAIT cells alone or after exposure to UN or INF B-LCLs. As expected, lower levels of CD161+ cells were found among IL-18R+ TCR Va7.2+ MAIT cells after exposure to INF B-LCLs compared to those MAIT cells alone or exposed to UN B-LCLs (**Figure S1C**). Surprisingly, we also observed that CD161 mean fluorescence intensity (MFI), a measurement of the mean expression levels per cell, was significantly decreased in MAIT cells exposed to INF B-LCLs (**Figure S1D**). These results suggested that changes in CD161 expression on MAIT cells was dependent on B cell interaction and decreases in their expression levels due to *S*. Typhi-infected B cells. Because previous studies have demonstrated that CD161 molecules modulate IFN-γ production by T cells (27–30), we hypothesized that CD161 molecules might play a similar role in MAIT cells. To test this assumption, PBMC were treated with 20 μg/ml of purified anti-CD161 neutralizing Abs, or isotype control, for 2 hours before co-culture with INF B-LCLs. After 16-18 hours of co-culture, cells and supernatants were collected. Using flow cytometric analyses, we observed that blocking of CD161 molecules decreased production of IFN-γ by MAIT cells exposed to INF B-LCLs as compared to MAIT cells exposed to INF B-LCLs treated with isotype (**Figure S1E**). To confirm and expand these observations on IFN-γ down-regulation, we measured IFN-γ in the culture supernatants. We found that CD161 blocking also reduced the release of IFN-γ into supernatants after co-culture with INF B-LCLs (**Figure S1F**). Of note, we cannot exclude that the smaller blocking effect observed in **Figure S1F** as compared to panel **Figure S1E** was due to the production of IFN-γ by TCRγδ cells and NK cells within the PBMC, which are known to produce IFN-γ but might not be blocked by anti-CD161 neutralizing Abs. Thus, we concluded that the expression levels of CD161 influence IFN-γ production by MAIT cells.

### LLT1 Induction on *S*. Typhi-Infected B-LCLs and Its Effect on MAIT Cell Function

**Figure 1:**
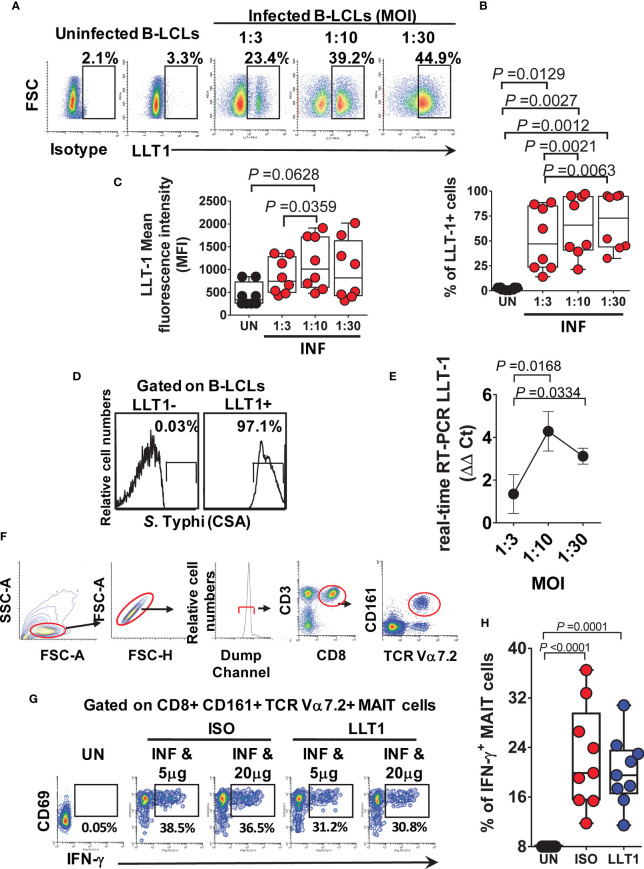
Effect of LLT1 expression on MAIT cell function. B-LCLs were infected with *S*. Typhi for 3 hours. Uninfected cells were used as controls. After 16-18 hours of gentamicin treatment, cells were stained with anti-LLT1 Ab and analyzed by flow cytometry. **(A)** Percentage of LLT1 on the surface of B-LCL cells. Cumulative LLT1 **(B)** percentage and **(C)** mean fluorescence intensity (MFI). Bar graphs extend from the 25^th^ to 75^th^ percentiles; the line in the middle represents the median of the pooled data. The whiskers delineate the smallest to the largest value. **(D)** *S*. Typhi infection levels in live LLT-1- and LLT1+ B-LCLs. **(E)** real-time RT-PCR for LLT-1. Data are representative of one independent experiment with 4 volunteers, three replicates each. **(F)** Representative gating strategy. **(G)** PBMC were exposed to either uninfected (UN) or *S*. Typhi-infected (INF) B-LCLs treated with different concentrations of Abs to LLT1 (LLT1) or isotype (ISO) control (5 and 20 μg/ml). After 16-18 hours of co-culture, cells were collected, and the levels of IFN-γ expressing MAIT cells were evaluated by flow cytometry. **(H)** Cumulative data of IFN-γ + MAIT cells after LLT1 blocking. Data are representative of two independent experiments.

It is well established that the LLT1/CD161 interaction inhibits NK cell-mediated cytotoxicity and IFN-γ secretion (25, 27, 28). Thus, we next investigated the effect of LLT1/CD161 interaction on MAIT cell production of IFN-γ. First, B-LCLs were infected with *S.* Typhi for 3 hours at different multiplicity of infection (MOI). Uninfected cells were used as controls. After 16-18 hours of gentamicin treatment, cells were stained with anti-LLT1 mAbs and analyzed by flow cytometry. We found that INF B-LCLs upregulated LLT1 expression on the cell surface in a dose-dependent manner, whilst UN B-LCLs expressed only negligible levels of LLT1 (**Figures 1A–C**). Virtually all LLT1+ B-LCLs were *S*. Typhi infected (**Figure 1D**). Real-time RT-PCR confirmed the upregulation of LLT1 (**Figure 1E**). To determine whether the same phenomenon was observed in primary autologous B cells, B cells were isolated by negative depletion to avoid activation and exposed to *S.* Typhi. As can be seen in **Figure S2**, primary autologous B cells (~90% pure) performed comparably to B-LCLs. Virtually all LLT1+ primary B cells were *S*. Typhi infected (**Figure S2E**). These results agree with previous studies showing that CpG and other TLR agonists induce LLT1 expression on B cells (24–27). Next, we investigated the effect of blocking LLT1 on MAIT cell functions. PBMC were exposed to either UN or INF B-LCLs treated with two different concentrations of a blocking polyclonal antiserum to LLT1 or isotype control (5 and 20 μg/ml). After 16-18 hours of co-culture, cells were collected, and MAIT cell expression of IFN-γ evaluated by flow cytometry. We found that although LLT1 blocking reduced the production of IFN-γ, these reductions were not as prominent as the one observed when blocking CD161 binding (**Figures 1F–H**). We hypothesize that at least part of the decrease of IFN-γ production by CD161 blocking was due to another signal provided by the B-LCLs.

### Effect of HLA-G Expression on MAIT Cell Function

**Figure 2:**
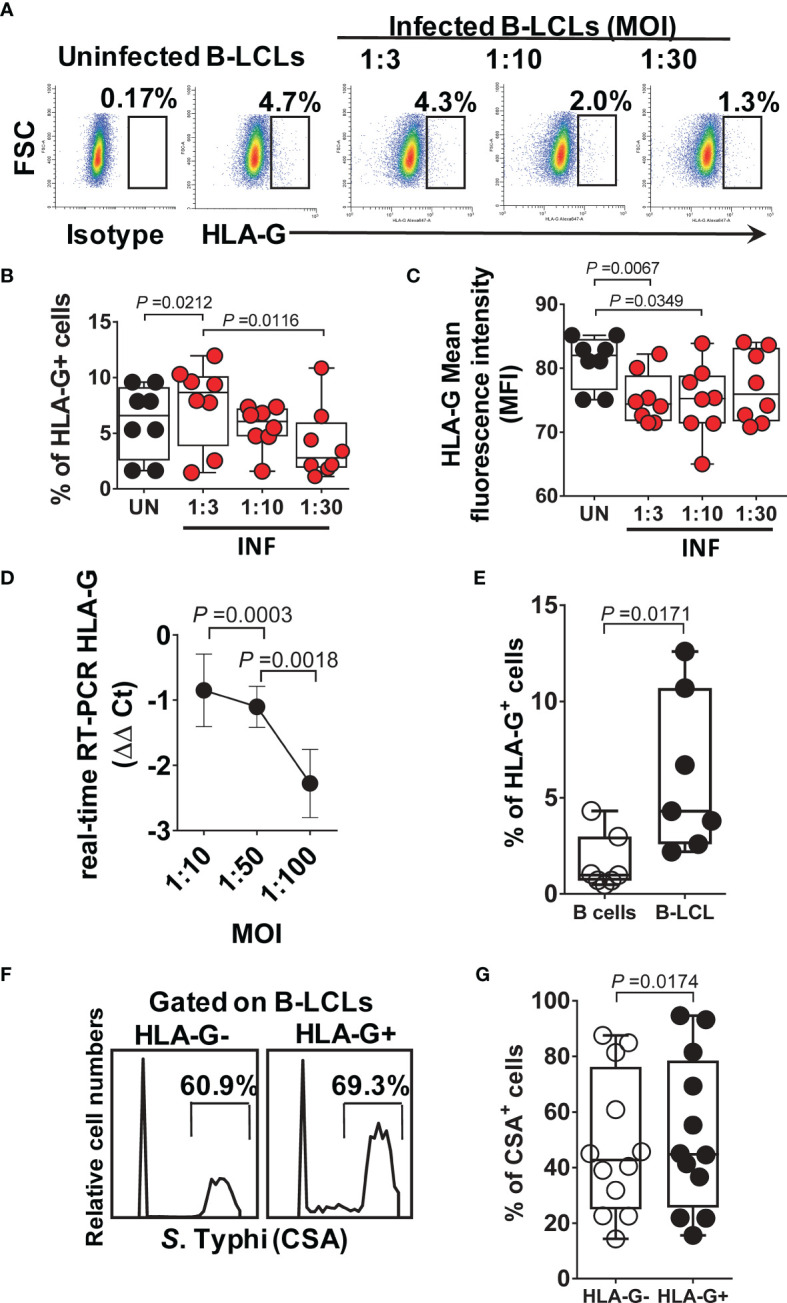
HLA-G expression on B cells. B-LCLs were infected with *S*. Typhi for 3 hours at different cell:bacteria multiplicity of infection (MOI). Uninfected cells were used as controls. After 16-18 hours of gentamicin treatment, cells were stained with anti-HLA-G mAbs and analyzed by flow cytometry. **(A)** Percentage of B-LCL cells expressing HLA-G on the surface. Cumulative HLA-G **(B)** percentage and **(C)** mean fluorescence intensity (MFI). Bar graphs extend from the 25^th^ to 75^th^ percentiles; the line in the middle represents the median of the pooled data. The whiskers delineate the smallest to the largest value. **(D)** real-time RT-PCR for HLA-G. Data are representative of one out of five independent experiments with one volunteer and six replicates. **(E)** HLA-G expression on B-LCLs from healthy individuals. Each dot represents one volunteer. **(F)** *S*. Typhi infection levels in live HLA-G- and HLA-G+ B-LCLs, Representative experiment. **(G)** Cumulative % of CSA+ cells. Data are representative of one out of six independent experiments with 4 volunteers and 2 replicates.

**Figure 3:**
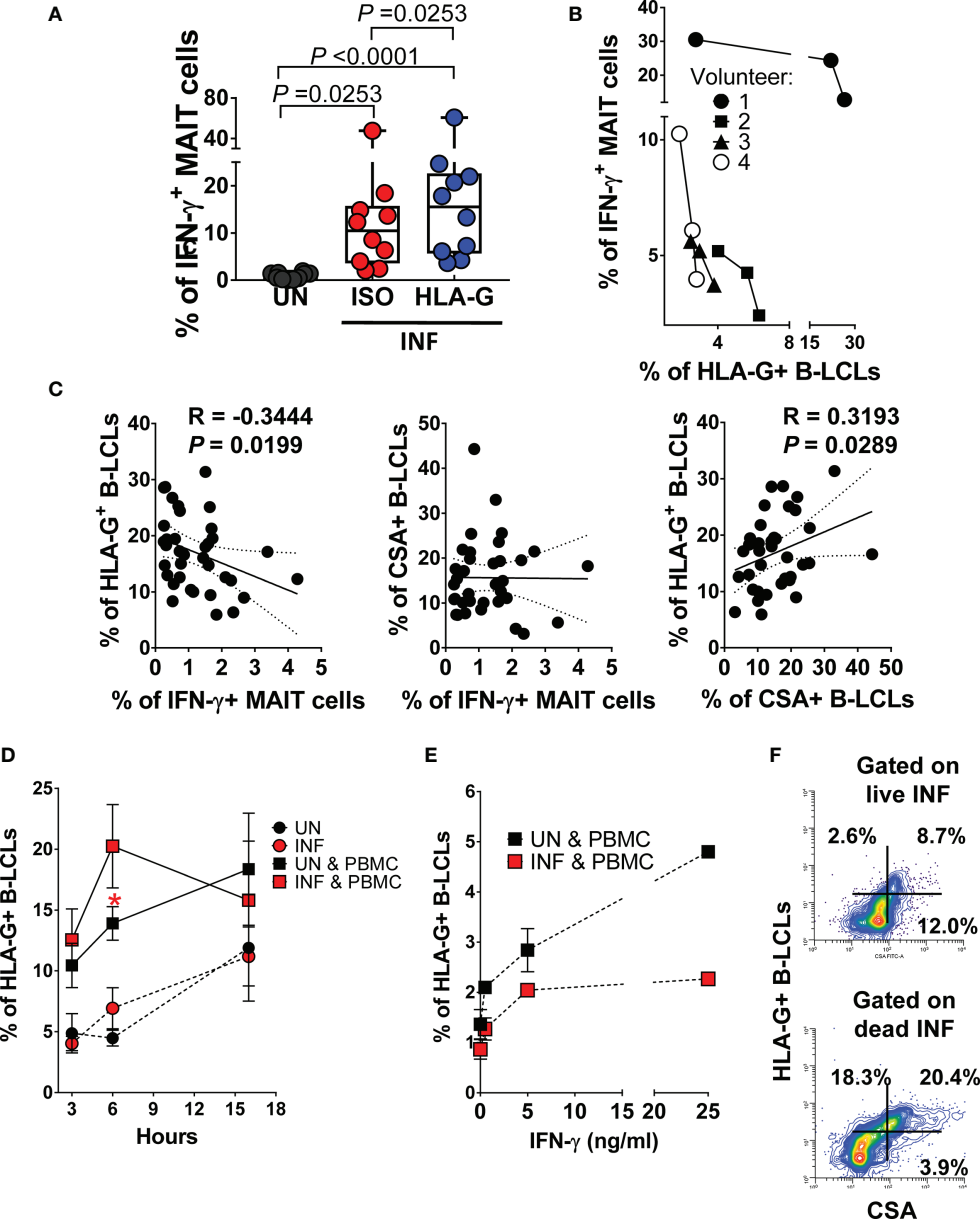
Effect of HLA-G expression on MAIT cell function. B-LCLs were infected with *S*. Typhi for 3 hours. Uninfected cells were used as controls. **(A)** PBMC were exposed to either uninfected (UN) or *S*. Typhi-infected (INF) B-LCL cells treated with Abs to HLA-G (HLA-G) or isotype (ISO) control (2.5 μg/ml). After 16-18 hours of co-culture, cells were collected, and the levels of MAIT cells expressing IFN-γ were evaluated by flow cytometry. Data are representative of two independent experiments. Bar graphs extend from the 25^th^ to 75^th^ percentiles; the line in the middle represents the median of the pooled data. The whiskers delineate the smallest to the largest value. **(B)** Relationship between the levels of IFN-γ+ MAIT cells and HLA-G expression on B-LCLs evaluated by mass cytometry. Four individual volunteers were studied under 3 different conditions (UN, ISO, and HLA-G). **(C)** Pearson’s correlations among levels of HLA-G and *S*. Typhi antigen (CSA) expression on B-LCLs and IFN-γ production by MAIT cells. Data are representative of at least five individual experiments. **(D)** Kinetics of HLA-G expression on B cells cultured alone (UN and INF) or cocultured with PBMC (UN & PBMC, and INF & PBMC). Data are representative of 3 individual experiments with 2-3 replicates Student’s t-test was performed to compare HLA-G+ B-LCL cell levels after exposure to MAIT cells. *, represent significant differences (*P*<0.05) between the uninfected (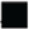) and infected B-LCLs (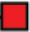) in the presence of MAIT cells at the same timepoint. **(E)** Dose effect of 3 concentrations of recombinant IFN-γ (0.5, 5, or 25ng/ml) on HLA-G expression on B cells cocultured for 18 hours with PBMC (UN & PBMC, and INF & PBMC). Co-cultures in the absence of IFN-γ (i.e., 0) were used as controls. Data are representative of 1 individual experiment with 2 replicates. **(F)** Expression of HLA-G *vs*. CSA on live (upper panel) and dead (lower panel) INF B-LCLs after 16-18 hours of co-culture with PBMC.

**Figure 4:**
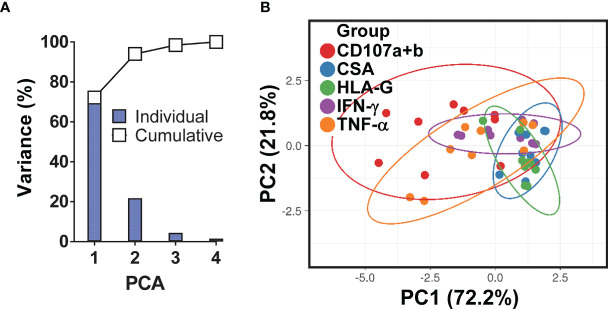
Principal Component (PC) Analysis variances. **(A)** The percent variations are plotted for each component (bars) and cumulatively (line). **(B)** Ability of PCA to cluster the levels of HLA-G and *S*. Typhi antigen (CSA) expression on B-LCLs and production of IFN-γ and TNF-α cytokines and expression of CD107a & b by MAIT cells. Unit variance scaling is applied to rows; singular value decomposition (SVD) with imputation is used to calculate principal components. X and Y axis show PC1 and PC2 that explain 72.2% and 21.8% of the total variance, respectively. Prediction ellipses are such that with probability 0.95, a new observation from the same group will fall inside the ellipse. Each dot represents a single experimental condition and volunteer, where each PC summarizes the variance of 75 data points for 3 experimental conditions (MAIT cells exposed to uninfected, or *S*. Typhi-infected B-LCLs that were treated with a neutralizing mAb to HLA-G (HLA-G), or isotype control (ISO) in 5 individual experiments).

Because HLA-G (**i**) inhibits NK and T-cell functions, and (**ii**) modulates IFN-γ expression (60), we evaluated whether HLA-G was involved in the inhibition of IFN-γ production by MAIT cells. To this end, B-LCLs were infected as described above. After 16-18 hours of gentamicin treatment, the HLA-G expression profile on B-LCLs was studied at the cell surface using the 87G mAb specific for the HLA-G1 and -G5 isoforms and flow cytometry. In the 2000 HLA-G/-E International Pre-workshop (Paris, France) (61), 87G was defined as a reference mAb to detect full-length membrane-bound and soluble HLA-G proteins. We found that the percentage of B-LCL cells expressing HLA-G on their cell surface decreased as a function of the infection level (**FigureS 2A–C**). The UN B-LCLs exhibited higher HLA-G expression on the cell surface than those observed in INF B-LCL (**Figures 2A–C**). These decreases were further confirmed at the transcriptional level by real-time RT-PCR (**Figures 2A–D**). Of note, whether testing primary B cells or B-LCLs, constitutive HLA-G expression was variable among UN from healthy individuals (**Figure 2E**). To determine whether the same phenomenon was observed in primary autologous B cells, B cells were isolated by negative depletion to avoid activation and exposed to *S.* Typhi. Primary autologous B cells behaved similarly to B-LCL (**Figure S3A**). *S.* Typhi-infected cells, as determined by CSA expression, was present in both HLA-G- and HLA-G+ B cell subsets, although the percentages of cells expressing CSA were significantly higher on HLA-G+ subset (**Figures 2F, G**, **S3B**). We next exposed PBMC to UN or INF B-LCLs treated with a neutralizing mAb to HLA-G or isotype control (2.5 μg/ml). Numerous studies have demonstrated a direct role of HLA-G antibodies in inhibiting immune responses by blocking the interactions between HLA-G and its receptors (62–65). After 16-18 hours of co-culture, cells were collected, and the production of IFN-γ in MAIT cells was evaluated by flow cytometry. Remarkably, in 4 out of 6 experiments, blocking the expression of HLA-G molecules on INF B-LCLs (**Figure 3A** **)** and autologous purified B cells (**Figure S3C** **)** resulted in increases in IFN-γ by MAIT cells. To confirm these results, we used mass cytometry to simultaneously study the levels of HLA-G and *S*. Typhi infection (CSA) on B-LCLs and the frequency of MAIT cells positive for IFN-γ, TNF-α, and CD107a+b (cytotoxicity marker). In 4 independents experiments, IFN-γ production by MAIT cells was inversely related to HLA-G expression levels on B-LCLs (**Figure 3B**). Indeed, we found a significant inverse correlation between HLA-G expression on B-LCLs and IFN-γ production by MAITs, and a direct correlation between HLA-G expression and the level of infection (CSA) on B-LCLs, but no correlation between CSA expression on B-LCLs and IFN-γ production by MAITs (**Figure 3C**). These remarkable results confirm our previous observations that the production of cytokines by MAIT cells was not dependent on bacterial antigen expression on B cells (9). Since HLA-G expression decreased in INF B-LCLs when cultured alone but a direct correlation between HLA-G and CSA is found when B-LCLs are co-cultured with MAIT cells, it is reasonable to hypothesize that to overcome the effects of *S*. Typhi infection, the host will upregulate HLA-G expression in B cells as a result of stimulation by IFN-γ secreted by MAIT cells. B cells are known to be able to express HLA-G when incubated with IFN-γ (60). Consequently, we measured the kinetics of HLA-G expression on B-LCLs after 3, 6, and 16 hours of co-culture with PBMC. B-LCL cells alone were used as a control. Co-inhibitory molecules vary in their expression kinetics; whereas some molecules are expressed at early stages, others are expressed at the late stages of activation (66). Regardless of the infection status, increased HLA-G expression became apparent 3 h after exposure to PBMC and was sustained for at least 6 h (**Figure 3D**). Importantly, when in the presence of PBMC, significant increases in the frequency of the HLA-G+ occurred 6 h after co-culture with INF B-LCLs as compared to UN counterpart (**Figure 3D**). To confirm and extend these results, we measured the HLA-G expression on B-LCLs after 16 hours of co-culture with PBMC in the absence or presence of 0.5, 5, or 25 ng/ml of human recombinant IFN-γ. Regardless of the infection status, B-LCLs increased HLA-G expression in function of the recombinant IFN-γ levels added. Of note, the effects of IFN-γ on HLA-G expression by B-LCL appear to be higher in UN than when cocultured with INF (**Figure 3E**). Interestingly, dead INF B-LCLs had higher HLA-G expression levels than live INF B-LCLs, suggesting that the increases in the frequency of HLA-G+ in UN were due to the loss of HLA-G+ INF B-LCLs (**Figure 3F**). Finally, Principal Component Analysis (PCA) was performed by ClustVis web tools (53) on the same data set. All replicates were merged for combined analysis, increasing the statistical power, and creating a matrix of 75 points. PC1 accounted for 72.2% of the total variance (**Figure 4A**), and analysis of its loadings shows tighter clustering of IFN-γ expression on MAIT cells, and CSA and HLA-G expression on B-LCL, as compared to TNF-α or CD107a+b expressing MAIT cells (**Figure 4B**).

### Inhibitory HLA-G Receptor CD85j and IFN-γ Secretion by MAIT Cells

**Figure 5:**
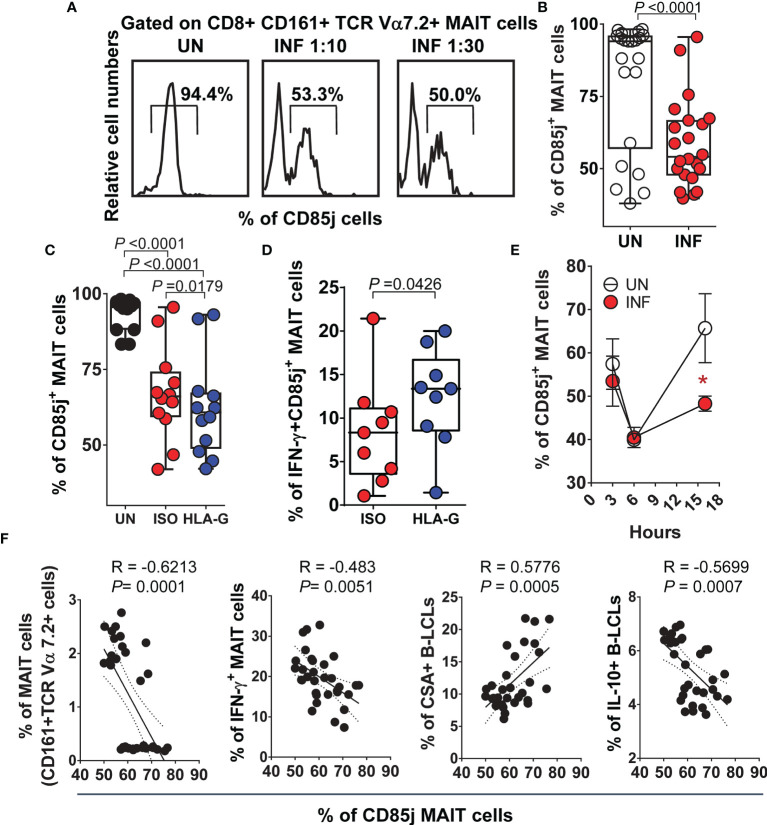
Inhibitory HLA-G receptor CD85j and IFN-γ secretion by MAIT cells. PBMC were exposed to either uninfected (UN) or *S*. Typhi-infected (INF) B-LCL cells treated with Abs to HLA-G (HLA-G) or isotype (ISO) control (2.5 μg/ml). After 16-18 hours of co-culture, cells were collected, and the levels of MAIT cells expressing CD85j and IFN-γ were evaluated by flow cytometry. To gate on MAIT cells, a “dump” channel was used to eliminate dead cells (ViViD^+^), as well as macrophages/monocytes (CD14^+^) and B-LCLs (CD45^+^) from the analysis. **(A)** Representative experiment showing CD85j expression on MAIT cells. **(B)** Cumulative data. Levels of MAIT cells expressing **(C)** CD85j only or co-expressing **(D)** IFN-γ after HLA-G blocking. **(E)** Kinetics of CD85j co-expression on MAIT cells after exposure to UN or INF B-LCLs. Student’s t-test was performed to compare CD85j expression on MAIT cells after exposure to UN or INF B-LCLs. *, represent significant differences (*P*<0.05) between MAIT cells exposed to UN (○) or INF B-LCLs (•) at the same timepoint. **(F)** Pearson’s correlations among levels of MAIT cells and their expression of CD85j and IFN-γ, and the expression of CSA and IL-10 on B-LCLs. Each dot represents a single experimental condition. Data are representative of one out of 2 individual experiments. Bar graphs extend from the 25^th^ to 75^th^ percentiles; the line in the middle represents the median of the pooled data. The whiskers delineate the smallest to the largest value.

Because HLA-G binds CD85j (also known as ILT2 receptor) expressed by some T and NK cells (67), we explored the role of CD85j in modulating the production of IFN-γ by MAIT cells. We exposed MAIT cells to UN or INF B-LCL treated with a neutralizing mAb to HLA-G or isotype control (2.5 μg/ml). After 16-18 hours of co-culture, cells were collected, stained with Abs to CSA, HLA-G, IL-10, IFN-γ, CD3, CD8, CD161, TCRα 7.2, and CD85j, and evaluated by flow cytometry. We found that the percentage of PBMC containing MAIT cells (**Figures 5A–C** **)** or sorted MAIT cells (**Figures S4A, B** **)** expressing CD85j on their cell surface decreased after infection. MAIT cells exposed to UN B-LCLs exhibited higher CD85j expression than those exposed to INF B-LCLs (**Figures 5A–C**, **S4A, B**). Interestingly, while HLA-G blocking resulted in decreases of CD85j+ MAIT cells, the concomitant expression of CD85j and IFN-γ was higher on MAIT cells exposed to INF B-LCLs treated with a neutralizing mAb to HLA-G compared to MAIT cells exposed to IFN B-LCLs treated with an isotype control (**Figures 5C, D**, **S4C**). We next evaluated the kinetics of CD85j expression on MAIT cells after 3, 6, and 16 hours of co-culture with UN or INF B-LCLs. Regardless of the infection status, decreased CD85j expression was observed 6 h after exposure to B-LCLs (**Figure 5E**). More importantly, significant increases in the CD85j+ MAIT cells occurred 16 h after co-culture with UN B-LCLs compared to the INF counterpart (**Figure 3D**). Finally, we found a significant inverse correlation between levels of CD85j and total MAIT cells and their levels of IFN-γ or the levels of IL-10 in B-LCLs (**Figure 5F**). We also found a direct correlation between CSA expression on B-LCLs and CD85j on MAIT cells (**Figure 5F**). Altogether, it is reasonable to speculate that although CD85j expression changes over time, as the infection progresses, B cells regulate the levels of CD85j and consequently IFN-γ production by MAIT cells.

### Effect of *S*. Typhi Infection on B Cell Function

**Figure 6:**
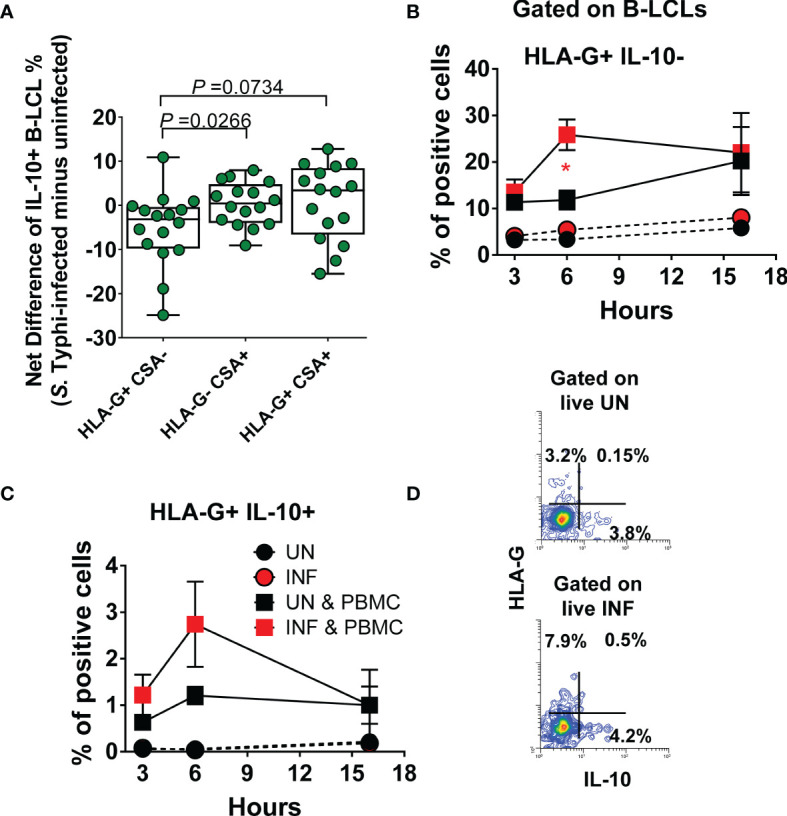
Effect of S. Typhi infection on B cell function. B-LCLs were infected with S. Typhi for 3 hours at 1:10 multiplicity of infection (MOI). Uninfected cells were used as controls. After 16-18 hours of gentamicin treatment, cells were co-cultured with PBMC. After 3, 6 and 16 hours of co-culture, cells were collected, and the B cell expression of CSA, HLA-G, and IL-10 were evaluated by flow cytometry. **(A)** Levels of IL-10 among B-LCL subsets (i.e., HLA-G+ CSA-, HLA-G- CSA+, and HLA-G+ CSA+). Bar graphs extend from the 25^th^ to 75^th^ percentiles; the line in the middle represents the median of the pooled data. The whiskers delineate the smallest to the largest value. Kinetics of **(B)** HLA-G+ IL-10- and **(C)** HLA-G+ IL-10+ expression on UN and INF B-LCLs alone or after exposure to PBMC. For **(B, C)** and “**C**”, Student’s t-test was performed to compare B-LCL subset levels after exposure to PBMC. *, represent significant differences (*P*<0.05) between the UN (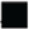) and INF B-LCLs (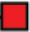) in the presence of PBMC at the same timepoint. **(D)** A presentative experiment showing the expression of IL-10 on UN and INF B-LCLs.

To gain further insight into the function of B cells expressing HLA-G, after 16-18 hours of gentamicin treatment, UN and INF B-LCLs were co-cultured with PBMC. After 3, 6, and 16 hours of co-culture, cells were collected, stained with Abs to CSA, HLA-G, IFN-γ, IL-10, CD3, CD8, CD161, and TCRα 7.2 and evaluated by flow cytometry. We used HLA-G and CSA markers to group B-LCLs into three subpopulations (i.e., HLA-G- CSA+, HLA-G+ CSA+, and HLA-G+ CSA-) and assessed their production of IL-10. When comparing the net differences (INF minus UN) among B-LCL subsets, the HLA-G+ CSA- subset had a significantly lower capacity to produce IL-10 than HLA-G- CSA+ and HLA-G+ CSA+ subsets (**Figures 6A, D**). We next investigated associations between the IFN-γ production by MAIT cells and expression of HLA-G, IL-10, and CSA by B-LCL subsets. We found a significant inverse correlation between the secretion of IFN-γ and HLA-G+ B-LCL, whether expressing CSA or IL-10 (**Table S1**). Of note, particularly striking inverse correlations were found between IFN-γ levels and the frequencies of the HLA-G+ IL-10+ B-LCL subset (*p <*0.001). These results support our hypothesis that IFN-γ secreted by MAIT cells upregulate HLA-G on B cells and that this upregulation is, at least in part, mediated by IL-10 production. Finally, we correlated the frequencies of MAIT cells with the expression of HLA-G and CSA on B-LCL subsets. Regardless of the HLA-G expression status, reductions in the frequencies of MAIT cells were inversely correlated with increases in CSA levels (**Table S2**). Remarkably, reductions in the frequencies of MAIT cells were directly associated with increases in the HLA-G+ CSA- B-LCL subset (**Table S2**). However, no statistically significant correlations were found between the frequencies of MAIT cells and the expression of HLA-G on total B-LCLs (**Table S2**). Finally, we measured HLA-G+ IL-10- and HLA-G+ IL-10-+ B-LCL subsets after 3, 6, and 16 hours of co-culture with PBMC containing MAIT cells. UN and INF B-LCLs alone were used as a control. Regardless of the infection status, the levels of both B-LCL subsets were higher in the presence of PBMC than when cultured alone (**Figures 6B, C**). More importantly, when in the presence of PBMC containing MAIT cells, significant increases in the frequency of the HLA-G+ IL-10- B-LCL occurred 6 h after co-culture with INF B-LCLs as compared to the UN counterpart (**Figure 6B**). We also observed that MAIT cells significantly decreased over a 16-hour exposure to INF as compared to UN B-LCLs (**Figure S5**).

## Discussion

Here, we provide compelling evidence supporting an association between the HLA-G expression on B cells and regulation of IFN-γ production by MAIT cells, suggesting that B cells can regulate MAIT cell function in humans. HLA-G is a critical immunomodulatory molecule, which belongs to the nonclassical HLA-class Ib molecules (68). HLA-G differs from classical HLA class I molecules by its genetic diversity, expression, structure, and functions. HLA-G has a very low amount of polymorphism, having only eight protein variants (*vs.* 462 for HLA-A and 789 for HLA-B). HLA-G was first identified as selectively expressed by choriocarcinoma cells (69–71). In fact, HLA-G expression is highly tissue-restricted. Besides being expressed in fetal tissues, such as trophoblast cells (72), HLA-G constitutive expression is only found in adult thymic medulla (73), cornea (74), pancreatic islets (75), and erythroid and endothelial-cell precursors (76). However, HLA-G expression can be induced in cancers (77, 78), transplantation (79), multiple sclerosis (80), inflammatory diseases (80–86), and viral infections (87, 88). HLA-G molecules can be present in seven different isoforms: four membrane-bound isoforms (HLA-G1, -G2, -G3, and -G4), and three soluble (s) ones (HLA-G5, -G6, and -G7). Likewise, soluble HLA-G1, which is structurally identical to soluble HLA-G5, can be generated by shedding membrane-bound HLA-G1 isoform through metalloproteases-mediated cleavage (68).

Based on our results, we propose a hypothetical feedback mechanism(s) of HLA-G expressing B cells regulating MAIT cell function (**Figure S6**). In this model, early during the infection, *S*. Typhi is endocytosed by B cells, leading to HLA-G downregulation on their surface. Infected B cells then present *S*. Typhi antigens together with MR1 molecules to TCR Vα7.2 on the MAIT cells membrane (1–3). After interacting with *S*. Typhi-infected cells (TCR Vα7.2-MR1, CD161-LLT1), activated MAIT cells respond to antigens by secreting TNF-α, IFN-γ, IL-17, granzyme a/b, and perforin (1, 7, 9, 89), the latter reinforcing their cytotoxic capability. As the infection progresses, B cells react to the increased IFN-γ secretion by upregulating the expression HLA-G (e.g., HLA-G1 & HLA-G5), leading to decreases on CD85j, which in turn limits the production of IFN-γ by MAIT cells, maintaining homeostasis, and avoiding dysregulated host responses. This hypothesis is supported by previous evidence that MAIT cells are known to trigger a harmful response in bacterial superantigen-mediated illness by contributing to cytokine storm pathology (90–92), and that B cells can express HLA-G and down-regulate IL-10 when incubated with IFN-γ (60, 93). Thus, sustained HLA-G inhibitory signals may trigger apoptosis of MAIT cells. Our previous results showed high levels of MAIT cells expressing CD57 and Caspase-3 markers in subjects after typhoid fever diagnosis (10). Similarly, Cerundolo’s group observed decreases in MAIT cell frequency during enteric fever after a controlled infection of humans with live *S*. Paratyphi A (11). Furthermore, previous reports showed that MAIT cells rapidly acquire the expression of exhaustion markers such as CD38, CD69, PD-1, and TIM in patients with acute viral infection by dengue or influenza (94). In the larger context, although central tolerance mechanisms delete the majority of autoreactive T-cells during a tightly controlled selection in the thymus, peripheral tolerance is required to avoid autoimmunity or aberrant immune responses during infections (95).

Another important finding was the increased expression of LLT-1 on B cells after *S*. Typhi infection. Activated B-cells are known to express the Lectin-like transcript-1 (**LLT1**) molecule, the ligand of CD161, on their membrane surface (24–27). However, blocking of LLT1 resulted in a minor, not statistically significant effect, suggesting the engagement of another receptor early during the infection. This hypothesis is supported by a previous study showing that, although LLT1 was found to be expressed on antigen-presenting cells, LLT1 blocking in the absence of antigen-presenting cells did not modulate the T cell activation thresholds and T cell effector functions (27). We postulate that the marginal LLT1 effect is due to kinetics issues. High expression of LLT1 was only achieved 16 hours after infection. As proposed by Braud and colleagues (27), a certain threshold of activation needs to be reached to get induction of LLT1 on the cell surface, which only occurs after 24 h of stimulation. It is also important to note that we tested only one polyclonal anti-LLT1 antibody. We cannot rule out that other anti-LLT1 neutralizing antibodies might perform better, thus further testing is warranted.

Several outstanding questions remain regarding the mechanism(s) of transcriptional and post-transcriptional regulation of HLA-G expression on MAIT cells. After *S*. Typhi exposure, MAIT cells undergo multiple coordinated modifications on Arg-me2(asy) and H4K20me3, H3K4me3, H4K20me1 epigenetic marks (47). Thus, epigenetic changes might be important in underlying these responses. Additionally, MAIT cells response to HLA-G bearing B cells might also be site-specific and governed by microenvironment factors. Previously, we (8, 96–98) and others (99) have shown that specific cell types are likely to acquire different phenotypes and functions in different microenvironments. In this regard, we observed that *S*. Typhi triggers gastric MAIT cell responses distinct from those activated by their counterparts in the periphery (8). It is unclear how these signals change in the gut, where MAIT cells are in frequent contact with the microbiota. Despite shared antigens, the microbiota may provide qualitatively and/or quantitatively different signals than pathogens (100). Future studies will be directed to address these important issues.

In conclusion, our findings that MAIT cell responses are regulated by HLA-G bearing B cells improve our understanding of the mechanisms by which B-cells control MAIT cell responses and might prove to be instrumental in the rational design of novel oral vaccines against *S*. Typhi and other enteric pathogens. MAIT cells are present at high basal levels in the human gut, and therefore they are attractive target cells to consider when developing novel oral vaccines and adjuvants. MAIT cells are located directly at the site of oral vaccine administration and the location of natural enteric pathogen infection. Thus, by modulating MAIT cells, it might be possible to promote/relieve gut inflammation directly, thereby affecting the outcome of oral vaccination. These current results will guide future investigations in the study of a new phenomenon, i.e., the study of regulatory mechanisms involving HLA-G in modulating MAIT cell responses to infections/pathogens. This understanding could lead to safer and more effective ways to therapeutically blocking or enhancing HLA-G expression to promote MAIT cell-mediated clearance of infected cells.

## Data Availability Statement

The original contributions presented in the study are included in the article/**Supplementary Material**, further inquiries can be directed to the corresponding authors.

## Ethics Statement

The studies involving human participants were reviewed and approved by University of Maryland Institutional Review Board protocol number HP-00040025. The patients/participants provided their written informed consent to participate in this study.

## Author Contributions

RS-G designed the study, performed the experiments, analyzed the data, and wrote the manuscript. TR, DL, and HT contribute to the design of the study, performed the experiments, analyzed the data, revised the manuscript, and approved the version submitted. MS contributed to the design, analyzed the data, and wrote the manuscript. All authors contributed to the article and approved the submitted version.

## Funding

This work was supported, in part, by the National Institute of Allergy and Infectious Diseases (NIAID), National Institutes of Health (NIH), Department of Health and Human Services (DHHS) federal research grants (https://www.niaid.nih.gov) R01 AI036525, U19 AI142725, and U19 AI082655 (Cooperative Center for Human Immunology [CCHI]) to MS. The funders had no role in study design, data collection, and analysis, decision to publish, or preparation of the manuscript.

## Author Disclaimer

The content is solely the responsibility of the authors and does not necessarily represent the official views of the National Institute of Allergy and Infectious Diseases, the National Institutes of Health.

## Conflict of Interest

The authors declare that the research was conducted in the absence of any commercial or financial relationships that could be construed as a potential conflict of interest.

## Publisher’s Note

All claims expressed in this article are solely those of the authors and do not necessarily represent those of their affiliated organizations, or those of the publisher, the editors and the reviewers. Any product that may be evaluated in this article, or claim that may be made by its manufacturer, is not guaranteed or endorsed by the publisher.
