# Supplementary material for: B Cells Control Mucosal-Associated Invariant T Cell Responses to Salmonella enterica Serovar Typhi Infection Through the CD85j HLA-G Receptor
Source: Front Immunol. 2021 Oct 1;12:728685. doi: 10.3389/fimmu.2021.728685 (PMC8517411; doi:10.3389/fimmu.2021.728685)
Supplement: Supplementary file 1 [file DataSheet_1.pdf]

Supplementary Information for

B cells control mucosal-associated invariant T cell responses to Salmonella enterica serovar Typhi *infection through the CD85j HLA-G receptor*

R. Salerno-Goncalves, T. Rezwan, D. Lou, H. Tettelin, and M.B. Sztein

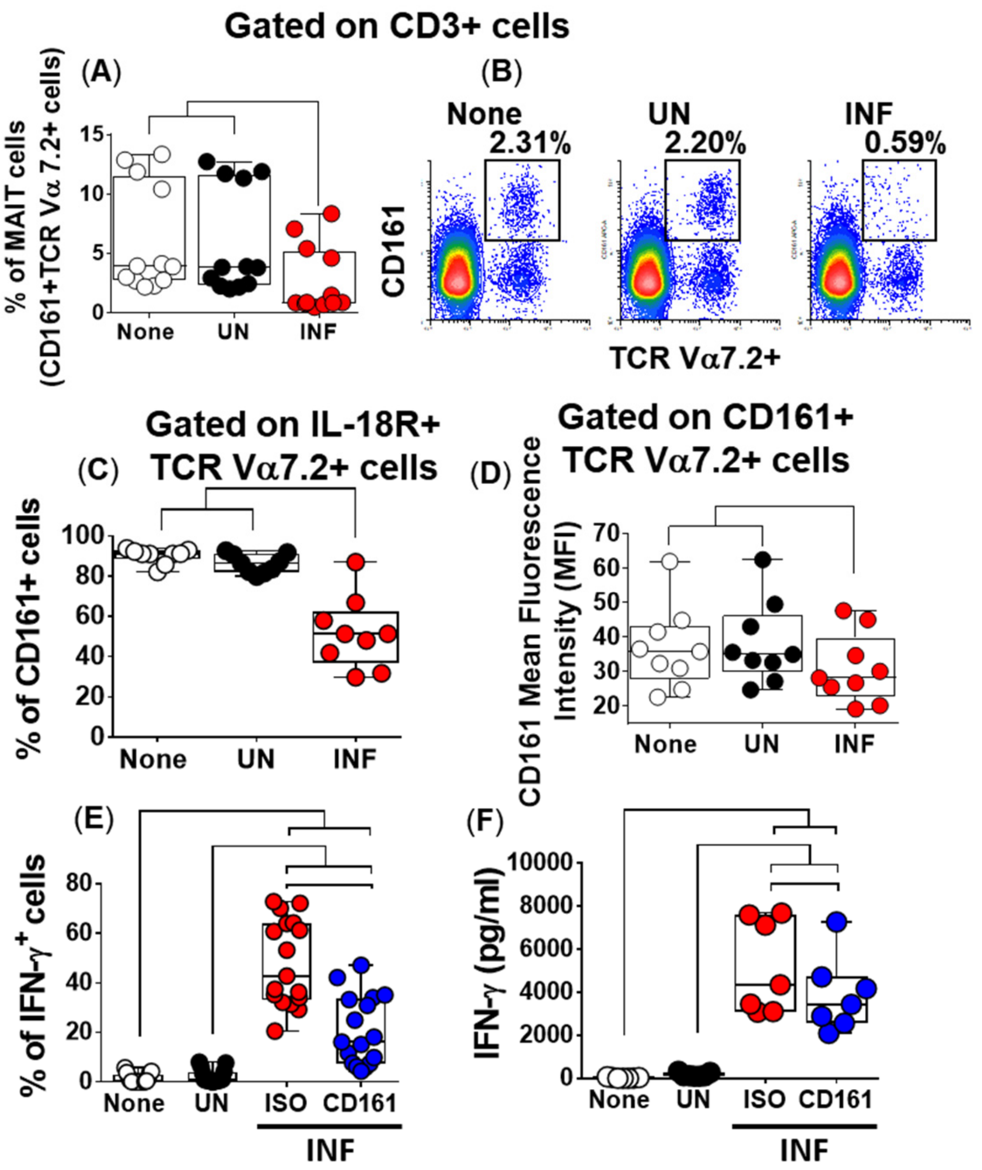

Fig S1. CD161 regulation of MAIT cell function. PBMC were exposed to either uninfected (UN) or *S*. Typhi-infected (INF) B-LCL cells. After 16-18 hours of co-culture, cells and supernatants were collected. (**A**) Percentage of MAIT cells alone (None) or after exposure to UN or INF B-LCLs were evaluated by flow cytometry; (**B**) cytograms of a representative experiment. (**C**) Percentage of CD161+ cells among IL-18R+ TCR Vα7.2+ MAIT cells. (**D**) Mean Fluorescence Intensity (MFI) of CD161 expressed on CD161+ TCR Vα7.2+ MAIT cells. (**E**) Percentage of MAIT cells secreting IFN-γ when alone or after exposure to INF B-LCLs. MAIT cells were treated with neutralizing mAbs to CD161 antigens (CD161), or isotype control (ISO), and exposed to either uninfected (UN) or *S*. Typhi-infected (INF) B-LCL cells. Single lymphocytes were gated based on forward scatter height vs. forward scatter area. A dump channel was used to eliminate dead cells (ViViD+) as well as macrophages/monocytes (CD14+), B lymphocytes (CD19+) and targets (CD45+) from analysis. This was followed by additional gating on CD3, CD8, TCR Vα7.2 and CD161 or IL-18R to identify MAIT cells, and then on IFN-γ to detect cytokine producing cells. These data represent 5 individual experiments. (**F**) IFN-γ in the culture supernatants were measured by using the Meso Scale Discovery (MSD) platform. The data represent 2 individual experiments. Horizontal lines represent significant differences (P<0.05) between the indicated culture conditions. Bar graphs extend from the 25th to 75th percentiles; the line in the middle represents the median of the pooled data. The whiskers delineate the smallest to the largest value.

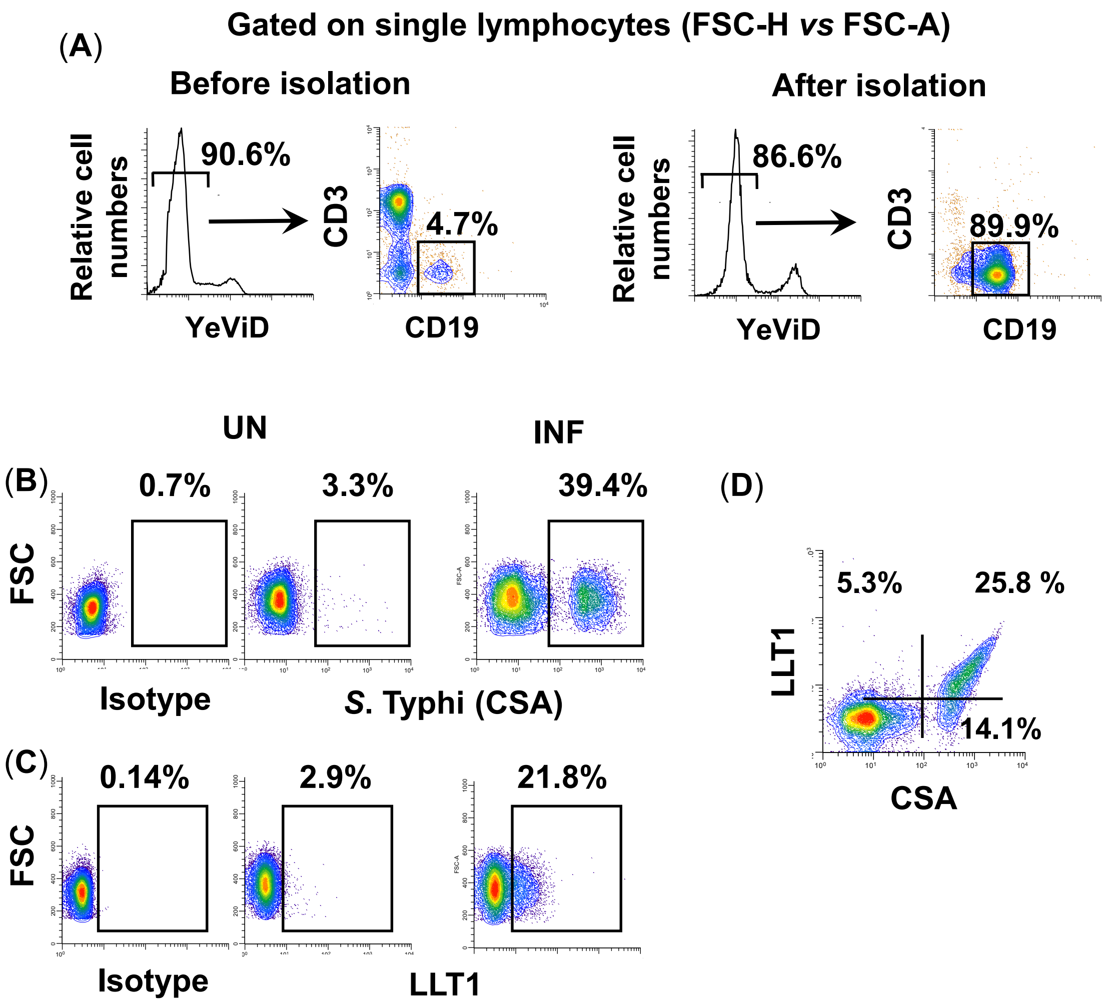

Fig S2. Isolation of primary B cells and evaluation of CSA and LLT1 markers. (**A**) Untouched primary human B cells were isolated from PBMC by negative selection using human B cell immunomagnetic beads. Isolated B cells were either left uninfected (UN) or were infected with *S*. Typhi (INF). After 16-18 hours of gentamicin treatment, cells were stained with anti-CSA (**B**) or LLT1 (**C**) Ab and analyzed by flow cytometry. (**D**) *S*. Typhi infection levels vs. LLT1 expression on B cells. These data represent 2 individual experiments with similar results.

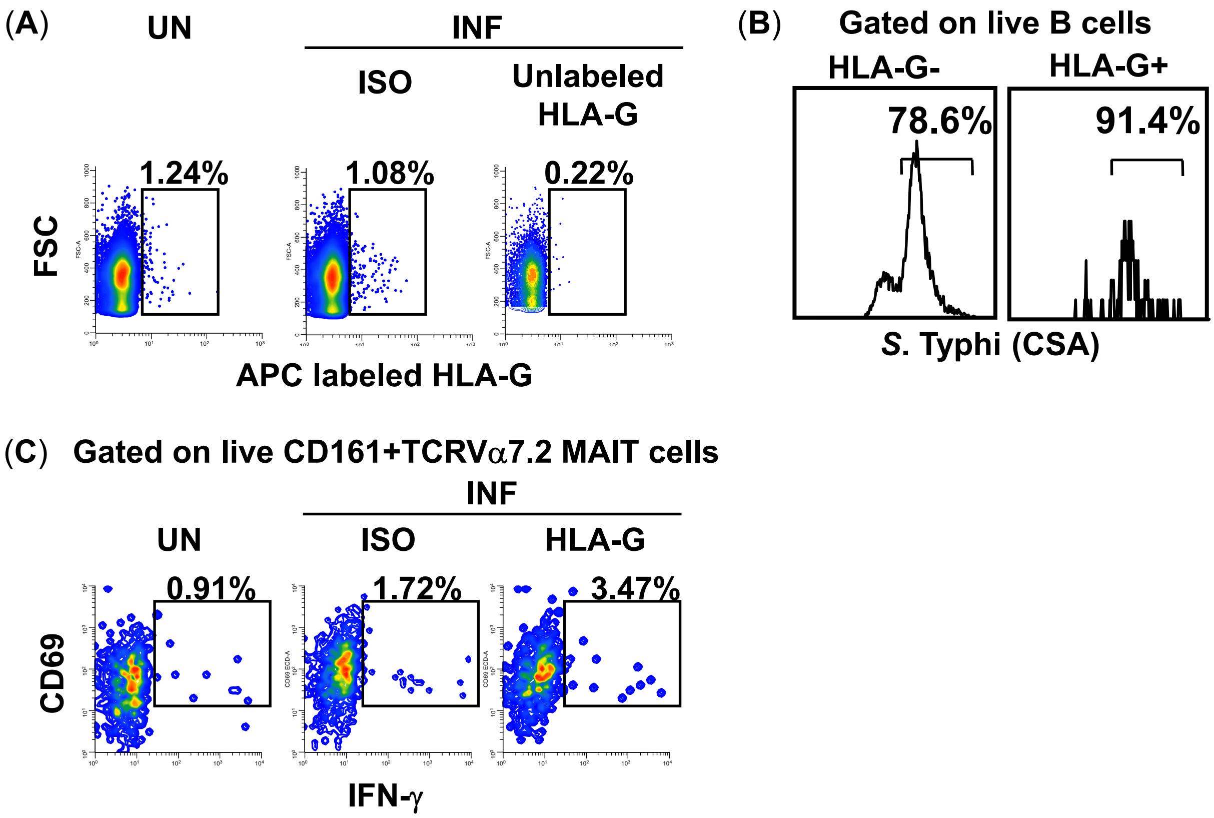

Fig S3. HLA-G expression on primary B cells and MAIT cells responses. Untouched primary human B cells were isolated from PBMC by negative selection using human B cell immunomagnetic beads. Isolated B cells were either left uninfected (**UN**) or infected with *S*. Typhi (**INF**). (**A**) After 16-18 hours of gentamicin treatment, cells treated with HLA-G neutralizing Ab (unlabeled HLA-G) or isotype (ISO) control (2.5 μg/ml) for 1 hour, and then stained with APC labeled HLA-G Ab and analyzed by flow cytometry. (**B**) *S*. Typhi infection levels in live HLA-G- and HLA-G+ B cells. These data represent 2 individual experiments with similar results. (**C**) PBMC were exposed to autologous B cells either left UN or infected with *S*. Typhi and treated with Abs to HLA-G (HLA-G) or isotype (ISO) control (2.5 μg/ml). After 16-18 hours of co-culture, cells were collected, and the levels of MAIT cells expressing IFN-γ were evaluated by flow cytometry. Data are representative of two independent experiments with similar results.

Table S1. Pearson's Correlations of IFN-γ produced by MAIT cells and HLA-G, *Salmonella* antigen (CSA) and IL-10 expression on B cells

Phenotype*		Number of	Pearson's Corr.	
MAIT cells	B cells	Observations	r	*P* value‡
Total IFN-γ+	Total HLA-G+	36†	-0.344	**0.020**
	HLA-G+CSA-	36	-0.371	**0.013**
	HLA-G+CSA+	36	-0.299	**0.038**
	HLA-G-CSA+	36	0.234	0.085
	HLA-G+IL-10-	36	-0.280	**0.049**
	HLA-G+IL-10+	36	-0.515	**<0.001**
	HLA-G-IL-10+	36	-0.049	0.388
	CSA+IL-10-	36	0.177	0.152
	CSA+IL-10+	36	-0.244	0.076
	CSA-IL-10+	36	-0.150	0.191

*Measured by Flow Cytometry

†Pooled data from 2 different experiments with 3 volunteers and 6 observations each

‡Bold fold represent significant correlations. The correlation coefficient "r" and "*P*" values are shown. *P* values of <0.05 were considered statistically significant

Table S2. Pearson's Correlations of MAIT cell frequencies and HLA-G and *Salmonella* antigen (CSA) expression on B cells

Phenotype*		Number of	Pearson's Corr.	
**MAIT cells**	**B cells**	**Observations**	**r**	***P* value‡**
Total	Total HLA-G+	36†	0.086	0.310
	Total CSA+	36	-0.529	**<0.001**
	HLA-G+CSA-	36	0.429	**0.005**
	HLA-G+CSA+	36	-0.319	**0.029**
	HLA-G-CSA+	36	-0.505	**<0.001**

*Measured by Flow Cytometry
†Pooled data from 2 different experiments with 3 volunteers and 6 observations each
‡Bold fold represent significant correlations. The correlation coefficient "r" and "*P*" values are shown. *P* values of <0.05 were

Table S3. Expression of HLA-G and IL-10 on B-LCLs alone or in the presence of PBMC

	**UN**				**INF**				**UN & PBMC**				**INF & PBMC**			
**Hours**	**1**	**2**	**3**	**4**	**1**	**2**	**3**	**4**	**1**	**2**	**3**	**4**	**1**	**2**	**3**	**4**
HLA-G+IL-10-*																
3	4.15	3.12	3.12	2.67	2.72	4.39	4.06	5.04	8.74	13.1	10.8	12.8	6.06	15.2	13.7	19.1
6	3.69	3.99	1.52	4.14	7.16	5.02	4.22	5.3	11.4	10.7	7.67	17.4	16.2	30.9	29.3	27
16	4.81		6.6	6.04	4.81		9.3	10.1	5.66	26.9	28.1		4.94	31.2	29.9	
HLA-G+IL-10+																
3	0.04	0.05	0.1	0.01	0.03	0.22	0.03	0.03	0.19	0.62	0.97	0.75	0.18	1.31	2.31	1.07
6	0.01	0.11	0.11	0.02	0.01	0.06	0.07	0.01	1.15	0.95	1.75	0.98	0.56	2.55	2.82	5.04
16	0.04		0.1	0.24	0.04		0.2	0.1	0.2	1.45	1.36		0	2.5	0.51	

*Measured by Flow Cytometry; results from cells obtained from PBMC of 4 different individuals (i.e., 1, 2, 3, and 4)
UN, uninfected, INF, *S.* Typhi-infected B-LCL cells

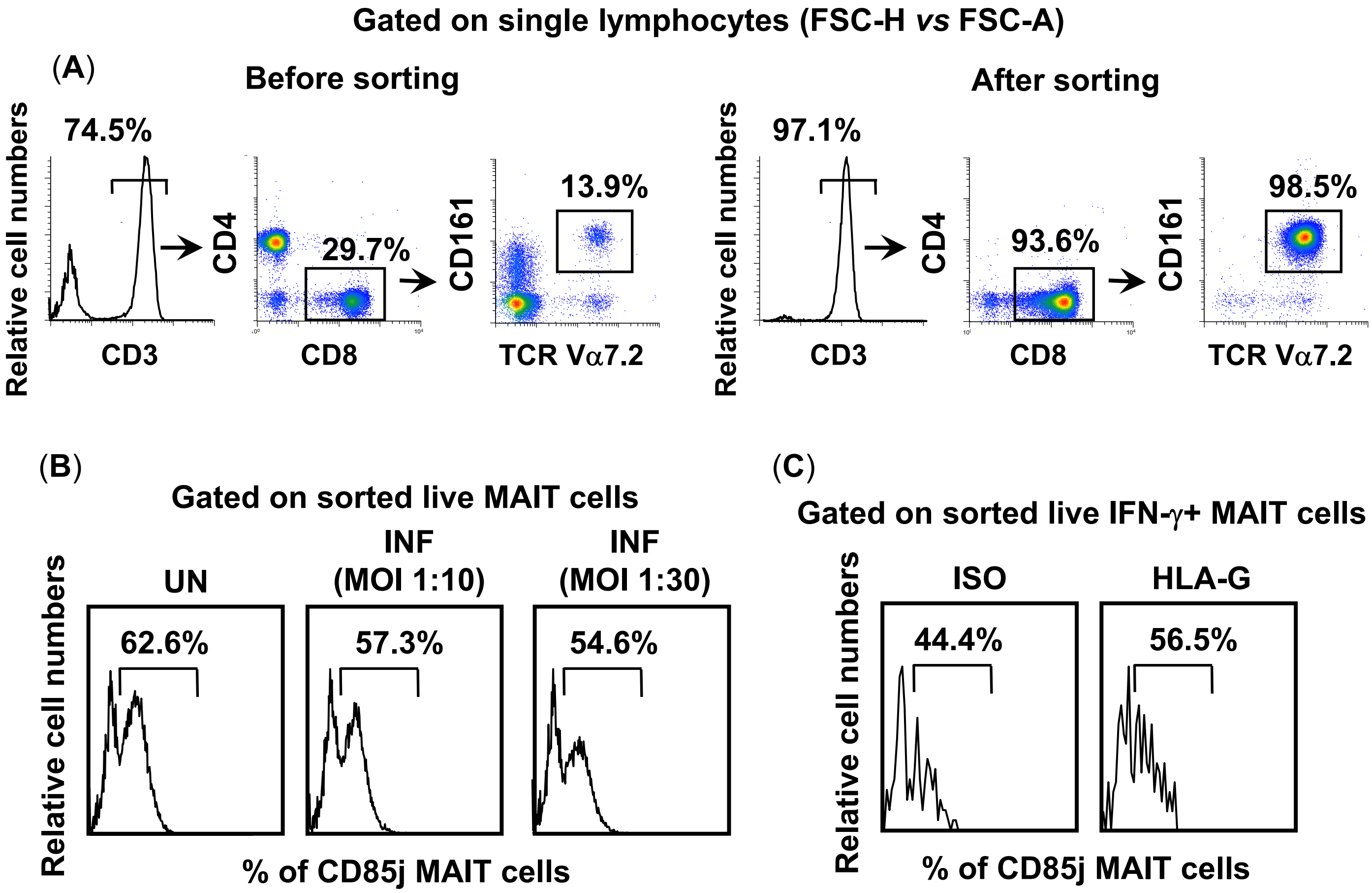

Fig S4. Sorting of MAIT cells and expression of the CD85j HLA-G inhibitory receptor after *S.* Typhi exposure. (**A**) MAIT cells (CD3+CD4-CD14-CD19-IL-18R+ TCR Vα7.2+) were sorted from PBMC. Sorted MAIT cells were exposed to either UN or INF B-LCLs treated with Abs to HLA-G (HLA-G) or isotype (ISO) control (2.5 μg/ml). After 16-18 hours of co-culture, cells were collected, and the levels of MAIT cells (CD3+CD4-CD14-CD19-CD161+TCRVα7.2+) expressing CD85j and IFN-γ were evaluated by flow cytometry. Representative experiment showing CD85j expression on sorted MAIT cells. Levels of MAIT cells expressing (**B**) CD85j only or (**C**) co-expressing IFN-γ.

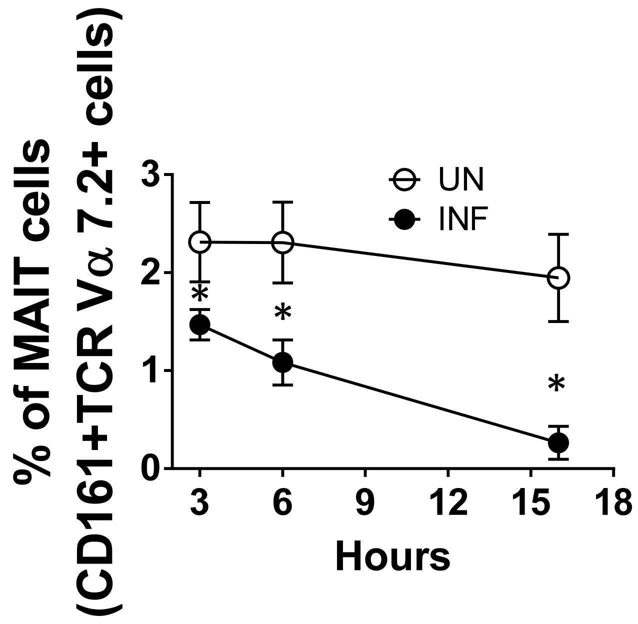

Fig S5. Kinetics of MAIT cell frequencies after exposure to *S*. Typhi-infected B-LCL. PBMC cells were exposed to either uninfected (UN) or *S*. Typhi-infected (INF) B-LCL cells. After 3, 6, and 16 hours of co-culture, cells were collected, and the percentages of MAIT cells were evaluate by flow cytometry. Error bars represent the SE of three biological replicates. Student's t-test was performed to compare MAIT cell levels after exposure to UN and INF B-LCL. *, represent significant differences ($P<0.05$) between the uninfected (○) and infected B-LCLs (●) at the same timepoint.

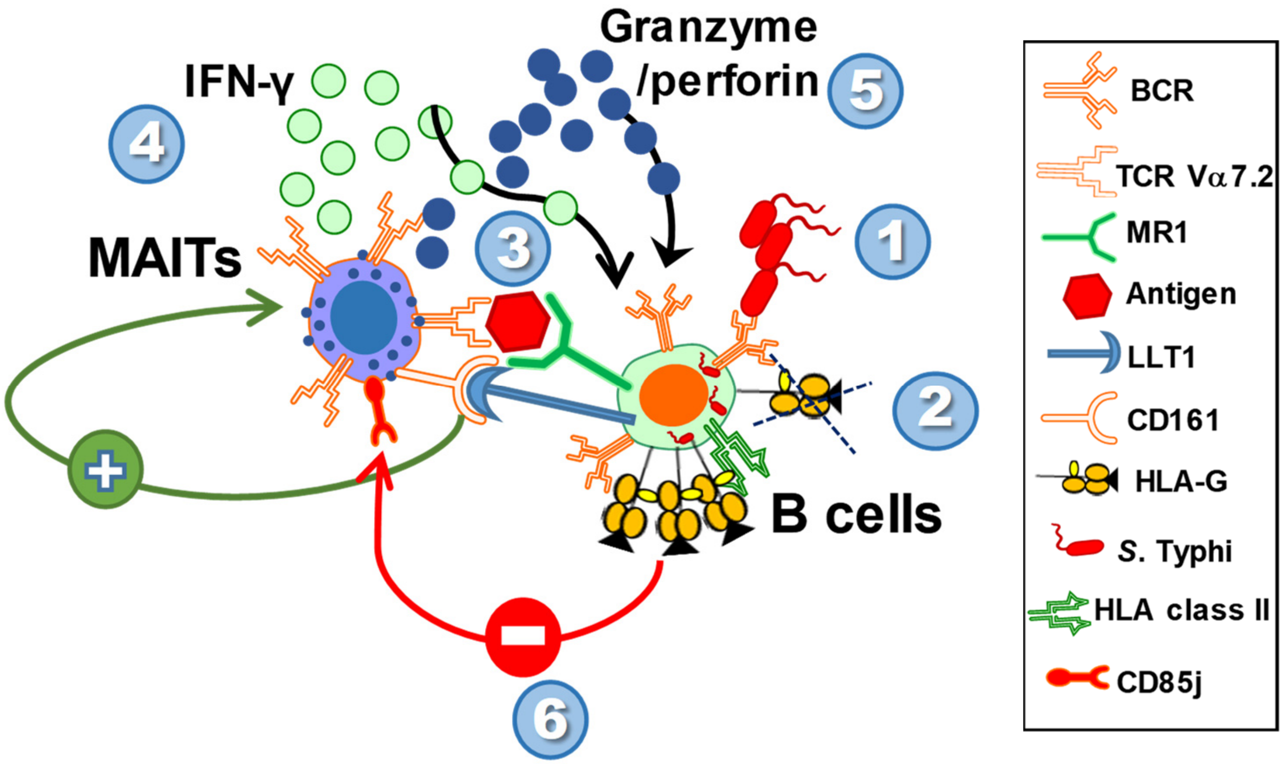

Fig S6. Hypothetical feedback mechanism(s) of HLA-G expressing B cells in the regulation of MAIT cell function. Early during infection, (**1**) *S*. Typhi are endocytosed by B cells, (**2**) triggering the downregulation of HLA-G on their surface. (**3**) Infected B cells present *S*. Typhi antigens together with MR1 molecules on the cell membrane to the TCR Vα7.2 on MAIT cells. After interacting with *S*. Typhi-infected B cells, activated MAIT cells secrete cytokines, including IFN-γ (**4**), and kill infected B-cells, likely through the (**5**) production of perforins and granzymes. As infection progresses, B cells respond to the increased IFN-γ secretion by (**6**) up-regulating the expression HLA-G (*e.g.*, HLA-G1 & HLA-G5), to limit the production of IFN-γ by MAIT cells through interaction with inhibitory HLA-G receptor CD85j on MAIT cells, thereby controlling inflammation to avoid dysregulated host responses. These inhibitory signals are likely to result in apoptosis of MAIT cells.
